# The Effect of Chlorpyrifos on Isolated Thoracic Aorta in Rats

**DOI:** 10.1155/2013/376051

**Published:** 2013-06-26

**Authors:** Ebru Yıldırım, Emine Baydan, Murat Kanbur, Oğuz Kul, Miyase Çınar, Hüsamettin Ekici, Nurgül Atmaca

**Affiliations:** ^1^Pharmacology and Toxicology Department, Veterinary Faculty, Kırıkkale University, Yahşihan, 71451 Kırıkkale, Turkey; ^2^Pharmacology and Toxicology Department, Veterinary Faculty, Ankara University, Dışkapı, 06110 Ankara, Turkey; ^3^Pharmacology and Toxicology Department, Veterinary Faculty, Erciyes University, Melikgazi, 38039 Kayseri, Turkey; ^4^Pathology Department, Veterinary Faculty, Kırıkkale University, 71451 Kırıkkale, Turkey; ^5^Pathology Department, Veterinary Faculty, Kyrgyzstan-Turkiye Manas University, 720044 Bishkek, Kyrgyzstan; ^6^Biochemistry Department, Veterinary Faculty, Kırıkkale University, 71451 Kırıkkale, Turkey; ^7^Physiology Department, Veterinary Faculty, Kırıkkale University, 71451 Kırıkkale, Turkey

## Abstract

This study investigated the effect of chlorpyrifos on thoracic aorta and on the level of NO in plasma and aorta. The effect of chlorpyrifos on thoracic aorta in organ bath was determined in 10 rats. Another 45 rats were assigned to 3 groups with 15 rats each: control group 1 received distilled water, control group 2 was given corn oil, and the last group was given 13.5 mg/kg chlorpyrifos dissolved in corn oil every other day for 8 weeks orally. Chlorpyrifos (10^−10^ M–10^−5^ M) showed no effect on isolated thoracic aorta. Plasma AChE activity was decreased, while LDH, ALT, GGT, and AST activities were increased in chlorpyrifos group compared to control groups. Plasma NO level was increased in chlorpyrifos group compared to control groups. iNOS expression was present in all groups in the cytoplasm of the endothelia and in the smooth muscle cells of aorta. According to semiquantitative histomorphological analysis, iNOS immunopositive reactions were seen in the decreasing order in chlorpyrifos, control 2, and control 1 groups. eNOS immunopositive reactions were observed in the endothelial cell cytoplasm, rarely in the subintimal layer, and the smooth muscle cells of aorta. There were no differences among the groups in terms of eNOS immunostaining. In conclusion, chlorpyrifos induced NO production in aorta following an increase in NOS expression.

## 1. Introduction

Organophosphates are anticholinesterase compounds, commonly used in veterinary medicine [1]. Chlorpyrifos is an organophosphorus insecticide that is widely used to control pests. It inhibits the acetylcholinesterase (AChE) enzyme, in the central and peripheral nervous systems [2, 3]. Chlorpyrifos was shown to cause lipid peroxidation [4, 5] and resultant oxidative stress may lead to reduced bioactivity of nitric oxide (NO) [6]. Nitric oxide is a free radical that contains an unpaired electron in the highest orbital. It is a highly reactive molecule [7]. Organophosphates cause excessive formation of citrulline, a marker of NO/nitric oxide synthase (NOS) and reactive nitrogen species generation [1]. Rajeswara Rao et al. [8] demonstrated that malathion, an organophosphorus insecticide, inhibits the NOS activity in the rat brain. Chang et al. [9] concluded that mevinphos, an organophosphorus compound, by accumulating acetylcholine in the rostral ventrolateral medulla may induce toxicity on the activation of the M_2_ subtype muscarinic receptors via NO produced by inducible nitric oxide synthase (iNOS). 

Cardiac risk factors and cardiovascular diseases disturb the endothelial function [6]. Çetin et al. [10] studied the effect of chlorpyrifos on the morphology and the function of the rabbit heart by echocardiography and they detected functional heart disorders induced by chlorpyrifos. Slotkin et al. [11] showed that chlorpyrifos caused cardiac autonomic input imbalance in neonatal rats. Ten and 25 mg/kg of chlorpyrifos cause an increase in systolic, diastolic, and mean blood pressure in 2-hour time in rats [12]. Also Smith and Gordon [13] suggested that chlorpyrifos elevated the blood pressure in spontaneously hypertensive rats. Yavuz et al. [14] observed that methidathion caused vascular wall damage and suggested that lipid peroxidation may be among the causative factors.

Although the organophosphates showed their mode of action primarily by inhibiting AChE, some of the organophosphates have been shown to bind membrane muscarinic receptors [15, 16]. An organophosphate compound named diisopropyl-fluorophosphate caused a concentration dependent contraction in rat aorta by the release of norepinephrine from adrenergic nerve endings [17]. There are studies about the oxidative stress in chlorpyrifos exposed animals [4, 5, 18–20], and there are also studies about the changes in NO caused by organophosphates [8, 9]. But so far a few limited studies have addressed the level of NO in the aorta in cases of chlorpyrifos exposure. Therefore, the first objective of this study was to determine the direct effect of chlorpyrifos, an organophosphorus insecticide, on the isolated thoracic aorta, and the second objective was to show chlorpyrifos induced changes in the level of NO in the plasma and the aorta and histopathological changes in thoracic aorta. 

## 2. Materials and Methods

In this study, fifty five, 250–300 g, 65–110-day-old healthy male Wistar rats were used. The rats were kept in cages at room temperature (25°C), allowed *ad libitum* access to standard rat diet and water under 12 h light/12 h dark cycle. The rats were also allowed to acclimatize to the animal facility for at least seven days before the start of the experiments. The study was conducted at the University of Kirikkale, Faculty of Veterinary Medicine, Experimental Animals Units. The animal care and use protocol was reviewed and approved by the Ethics Committee of the Faculty of Veterinary Medicine, Kirikkale University (30.04.2007-07/06).

Two sets of experiments were performed in this study. In the first set, 10 rats were used to show the direct effect of chlorpyrifos on isolated rat thoracic aorta. The rats were sacrificed under thiopental sodium (Pentothal Sodium, Abbott, Turkey, 50 mg/kg) anesthesia and the thoracic aorta was removed. Ring segments (4 mm length) were prepared in Krebs-Henseleit solution (composition in mM: NaCl: 118, KCl: 4.7, MgSO_4_: 1.2, KH_2_PO_4_: 1.2, NaHCO_3_: 25, CaCl_2_: 2.5, and D-Glucose: 10.6). The aortic rings were mounted between two wire hooks and suspended in organ baths containing 10 mL Krebs-Henseleit solution, maintained at 37°C, and bubbled with 95% CO_2_ and 5% O_2_ to keep the pH at 7.4. The aortas were washed in 15-minute (min) intervals. After 60 min of equilibration under the resting tension of 2 g, the aortas were contracted with 10^−6^ M phenylephrine (dissolved in distilled water) and washed 3 times in 15-minute intervals. Then cumulative chlorpyrifos concentrations (10^−10^ M, 3 × 10^−9^ M–10^−5^ M) were applied to the aortic rings. Chlorpyrifos is not practically soluble in water, so it was firstly dissolved in acetone, and this dilution (0.1 M chlorpyrifos) kept as a stock solution. Other chlorpyrifos dilutions were made with distilled water [21]. In another organ bath the aortic rings were precontracted with submaximal dose of phenylephrine (10^−6^ M) and cumulative chlorpyrifos concentrations (10^−10^ M, 3 × 10^−9^ M–10^−5^ M) were applied to the aortic rings. As chlorpyrifos was dissolved in acetone, after washing the tissues 3 times in 15 min intervals, the same procedure was repeated with the same concentrations of acetone diluted with distilled water. The responses were expressed as percentage relaxation from phenylephrine precontraction. Data were measured with a force displacement transducer (FDT 05 MAY, COMMAT, Turkey) and recorded by the BIOPAC System, (MP35, USA).

 In the second set of the experiment, the rats were assigned to 3 groups with 15 rats each. The first group was the control 1 group which is given only distilled water, the second group was given corn oil (control 2), and the third group was given 13.5 mg/kg [4] chlorpyrifos dissolved in corn oil every other day for 8 weeks by an oral gavage. At the end of the experiment, the animals were fasted overnight and sacrificed under thiopental sodium anesthesia, and blood samples were taken from the hearts of the rats of each experimental group. The blood samples, collected into heparinized test tubes, were centrifuged at 3000 rpm for 10 min at 4°C. Plasma was collected and stored at −80°C for NO and biochemical analysis. Plasma and tissue NO levels were determined by the Griess method.

Acetylcholinesterase (EC 3.1.1.7), aspartate transaminase (AST) (EC 2.6.1.1), alanine transaminase (ALT) (EC 2.6.1.2), lactate dehydrogenase (LDH) (EC 1.1.1.27), creatine kinase (CK) (EC 2.7.3.2), gamma glutamyl transferase (GGT) (EC 2.3.2.2), total protein (TP), and albumin (ALB) levels were all determined spectrophotometrically (Shimadzu UV 1700, Japan) by commercial kits (TECO, CA, USA). Tissue NO level was measured by the modified Griess method [22]. 

After systemic necropsy procedure, 10 mm thoracic aorta samples were fixated in 4% buffered solution for 48–72 hours. Routine pathologic tissue follow-up procedure was applied as follows: aortas were rinsed in alcohol (70%, 80%, 90%, 96%, and absolute alcohol) and in xylol, respectively, and embedded in paraffin. Serial sections were cut at a thickness of 4-5 *μ*m, mounted on glass slides, and stained with hematoxylin and eosin for histopathological examination. For Masson's trichrome staining, commercial kits were used (Bio-Optica, Italy). For immunoperoxidase examinations to detect endothelial nitric oxide synthase (eNOS) and iNOS expessions in tissue sections, commercial eNOS and iNOS antigen specific monoclonal primary antibodies (NeoMarkers Fremont, CA, USA) and a commercial streptavidin/biotin immunoperoxidase kit (LSAB 2 system, HRP, DacoCytomation, Denmark) were used. 

Results were evaluated under binocular light microscope. iNOS and eNOS immunoreactivities in aorta sections were scored semiquantitatively as follows: 0: none; 1: very weak, 2: weak, 3: moderate, 4: dense, and 5: very dense.

### 2.1. Statistical Analysis

The data were expressed as arithmetic means and standard error (X ± SEM). Statistical analyses were performed by SPSS 15.0 version for Windows (SPSS Inc., Chicago, IL, USA). Paired sample *t*-test was performed to estimate the statistically significant difference between the % relaxation of acetone and chlorpyrifos concentrations precontracted with phenylephrine. One-way ANOVA test was used for biochemical and nitric oxide analyses. If the *F* values were significant, Duncan's Multiple Range Test was performed. Differences were considered as significant when the *P* value was less than 0.05.

## 3. Results

### 3.1. Pharmacological Results

**Figure 1:**
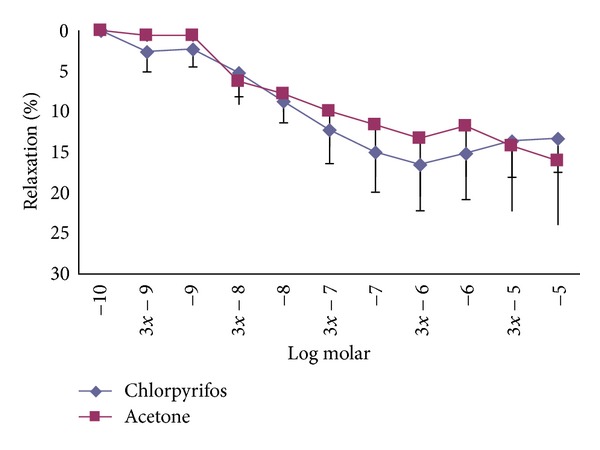
The % relaxation of chlorpyrifos and acetone in the thoracic aorta precontracted with 10^−6^ M phenylephrine.

Cumulative chlorpyrifos concentrations (10^−10^ M, 3 × 10^−9^ M–10^−5^ M) had no effect on thoracic aorta of the rats alone. Also there was no statistically significant difference between the % relaxation of acetone and chlorpyrifos concentrations precontracted with phenylephrine (Figure 1).

### 3.2. Biochemical Results

The AChE enzyme activity and biochemical parameters in the plasma were presented in Table 1. The AChE enzyme activity was significantly decreased (*P* < 0.01), and the activities of LDH, ALT, GGT (*P* < 0.01), and AST (*P* < 0.05) were significantly increased in chlorpyrifos group as compared to control groups. Plasma CK activity in chlorpyrifos and corn oil group was increased insignificantly (*P* = 0.07). Plasma TP and albumin levels did not change among the groups (*P* > 0.05).

### 3.3. Pathological Results

#### 3.3.1. iNOS Expression

**Figure 2:**
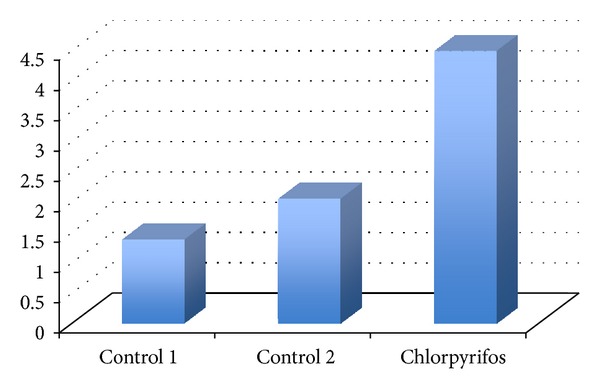
The semiquantitative scoring of iNOS expression in the aorta tissue sections, stained with iNOS primary antibody, ABC immunoperoxidase test. 1: very weak, 2: weak, 3: moderate, 4: dense, and 5: very dense.

iNOS expression was seen in all groups at the smooth muscle cell cytoplasm of the tunica media of the aorta. The order of density for immunopositive reactions was chlorpyrifos > control 2 > control 1 (Figure 2). The semiquantitative results for iNOS expression in the aorta tissue was given in Figure 2 for comparison purposes. 

**Figure 3:**
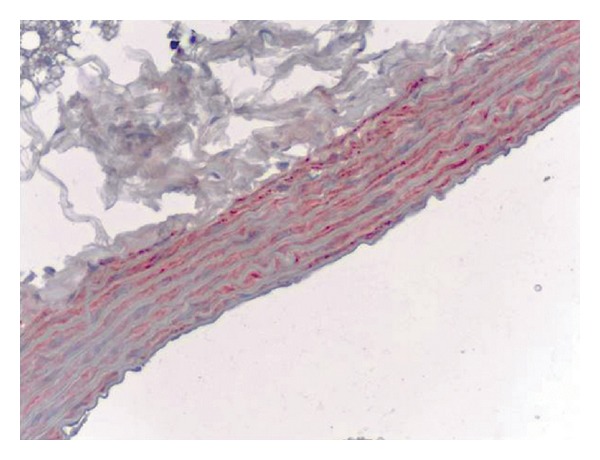
Control group (control 1), iNOS immunoreactivity in the rat aorta, monoclonal anti-iNOS antibody, ABC immunoperoxidase technique, Mayer's hematoxylin staining.

**Figure 4:**
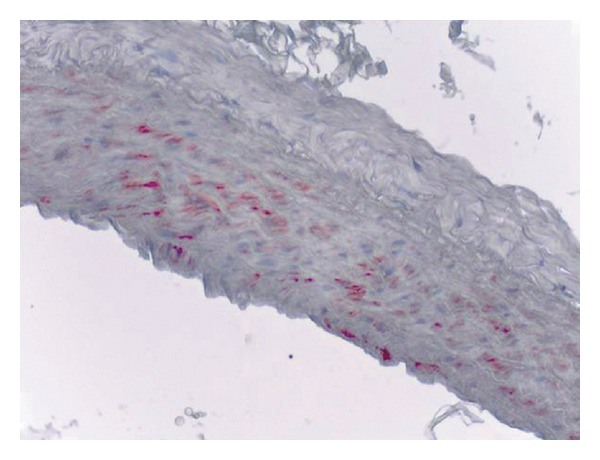
Corn oil group (control 2), iNOS immunoreactivity in the rat aorta, monoclonal anti-iNOS antibody, ABC immunoperoxidase technique, Mayer's hematoxylin staining.

**Figure 5:**
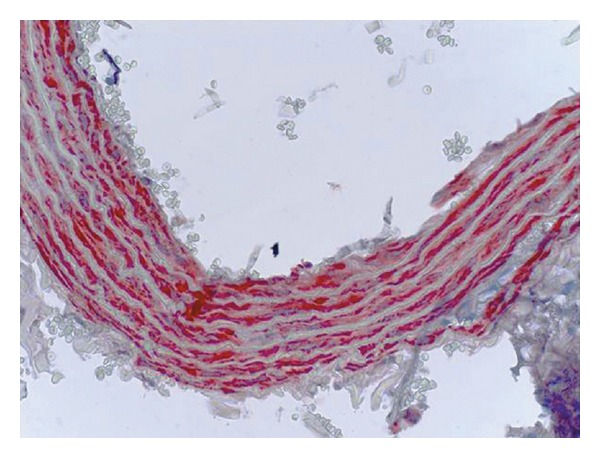
Chlorpyrifos group, iNOS immunoreactivity in the rat aorta, monoclonal anti-iNOS antibody, ABC immunoperoxidase technique, Mayer's hematoxylin staining.

Immunoreactions were seen in the vasa vasorum which is located between the aorta wall and the adventitia and also at the tunica media. There were no significant reactions at adventitial connective tissues and their cells (Figures 3, 4 and 5). 

#### 3.3.2. eNOS Expression

**Figure 6:**
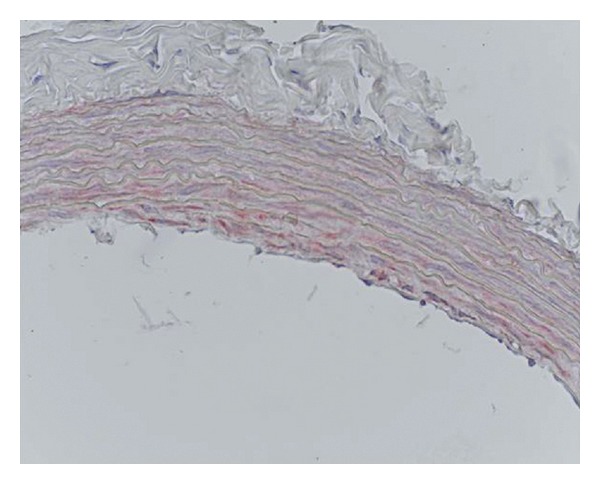
Chlorpyrifos group, eNOS immunoreactivity in the rat aorta, monoclonal anti-eNOS primer antibody, ABC immunoperoxidase technique, Mayer's hemotoxylin staining.

eNOS immunopositive reactions were seen in the endothelial cell cytoplasm of the tunica intima and rarely in the subintimal layer of the aorta. eNOS immunopositive reactions were also seen in the smooth muscle cells of aorta in the control (*n* = 4) and the chlorpyrifos groups (*n* = 1) (Figure 6). There were no differences between the groups in terms of eNOS immunostaining.

### 3.4. Nitric Oxide Results

The nitric oxide levels were increased in the thoracic aorta and the plasma of chlorpyrifos group as compared to control groups (Table 2).

## 4. Discussion

Cumulative chlorpyrifos concentrations (10^−10^, 3 × 10^−9^ M–10^−5^ M) had no effect on the thoracic aorta of rats in this study. Çetinkaya and Baydan [21] showed that cumulative concentrations of chlorpyrifos (10^−10^ M–10^−6^ M) caused contractions in rat intestinal muscle. This difference can be attributed to the receptor difference between the aorta and the ileum. Chlorpyrifos is a cholinesterase inhibitor with mainly muscarinic and nicotinic activities. On the other hand in the thoracic aorta the contractions primarily occur via alpha-1 receptor stimulation [23]. Lim et al. [17] found that diisopropyl-fluorophosphate contracted the rat aorta in a concentration dependent manner and suggested that this contraction was mediated by norepinephrine released from sympathetic nerve terminals. Similar to our study, Ebeigbe and Campbell [24] showed that dichlorvos, an organophosphate insecticide, had no effect on baseline tension but relaxed norepinephrine, 5-hydroxytrytamine, and KCl contractions dose dependently on isolated rat tail arteries. In our study chlorpyrifos concentrations did not show an effect on the thoracic aorta precontracted with phenylephrine. Gordon and Padnos [12] demonstrated an increase in blood pressure in the unrestrained rat exposed to chlorpyrifos. So hypertension induction by chlorpyrifos probably is caused by other mechanisms of chlorpyrifos. 

Alanine transaminase, AST, GGT, and LDH enzyme activities are known to indicate the hepatic damage [25]. Mansour and Mossa [26] showed that the levels of AST, ALT, LDH, and GGT activities were increased in rats given chlorpyrifos. They suggested that the increase in the levels of these enzymes is secondary to a functional damage in the liver or to changes in the membrane permeability. Ncibi et al. [27] indicated that the increase in ALT, AST, and LDH enzyme activities in Swiss mice given chlorpyrifos can be attributed to cell membrane damage of the liver, and they discussed that, because of this damage, these enzymes were passed to the blood circulation from the cytosol. In our study the enzyme activities of the liver were increased. These results were in agreement with the other studies conducted on chlorpyrifos [26–28].

It was indicated that chlorpyrifos suppresses the AChE activity in different tissues like liver, kidney, and spleen [29]. Plasma AChE activity is also suppressed by chlorpyrifos in rats, which can be attributed to the binding of chlorpyrifos to AChE [30]. Weilgomas and Krechniak [31] observed that 10 mg/kg chlorpyrifos caused 8.3% and 10% inhibition of AChE in rats on the 14th and the 28th days, respectively. Also in this study AChE enzyme activity was decreased in the chlorpyrifos group as compared to the control groups (*P* < 0.01).

Yavuz et al. [14] showed that organophosphorus insecticide methidathion caused vascular wall damage in rats. Guvenc Tuna et al. [32] detected that chronic chlorpyrifos administration for 90 days decreased the strength of the aorta. They suggested that this decrease may affect the response of the aorta to mechanical loading caused via blood pressure. In this study, though there were no prominent histopathological changes in the media or adventitia of aorta, endothelial hypertrophy and extended and roughened subintimal layer were observed in animals given chlorpyrifos. In this group of animals, eNOS and iNOS expressions also showed colocalization with damaged endothelial and subintimal cell cytoplasms. eNOS immunopositive reactions were also seen in the media layer of the smooth muscle cell cytoplasms as well. These results suggest that chlorpyrifos induced NO production in the damaged cells and tissues of the aorta following an increase in eNOS and iNOS expressions in the chlorpyrifos group. 

In our study the NO level in the plasma was significantly higher in chlorpyrifos group than the control groups. Similar to our study, Zhou et al. [33] showed that the plasma NO was significantly increased in patients with acute organophosphorus pesticide poisoning. Alp et al. [34] found that organophosphorus insecticide malathion elevated the NO levels, depending on the severity of the tissue damage. On the other hand, Soltaninejad et al. [35] found no difference in plasma total NO levels in acute exposure organophosphorus poisoning in humans.

Kim et al. [36] showed that diisopropyl-fluorophosphate administration caused severe limbic seizures and early necrotic and delayed apoptotic brain injuries, and they also observed that nitrite/nitrate content in the cerebrospinal fluid was elevated after 2 hours, and the maximum level was detected at 6th hours. Kanbur et al. [37] declared that propetamphos significantly increased the NO and other lipid peroxidation parameters in mice when given in the diet for 60 days. In this study the NO level in the aorta and the plasma was also increased in group given chlorpyrifos. Acetylcholine is known to induce NO synthesis via NOS in endothelial cells [38]. Exposure to bacterial polysaccharides, cytokines, and some xenobiotics leading to NO synthesis via iNOS in vascular smooth muscle causes excessive amount of NO production. Nitric oxide is a free radical able to cross cell membranes easily, and unlike other radical species, nitric oxide can diffuse to greater distances due to its high stability [39–41]. The results of the study showed that chlorpyrifos inhibited the AChE and led to accumulation of acetylcholine, triggering the synthesis of NO mediated by eNOS and iNOS. This NO easily passes to the circulation. 

## 5. Conclusions

As a result 13.5 mg/kg chlorpyrifos given for 8 weeks every other day increased the NO level in both plasma and aorta and decreased the AChE enzyme activity in the plasma. eNOS immunopositive reactions were seen mostly in endothelial cell cytoplasm of aorta (intima layer and rarely in the subintimal layer). Also in 4 samples of control group and 1 sample of chlorpyrifos group, eNOS immunopositive reactions were demonstrated at the media layer smooth muscle cell cytoplasm. Inducible NOS expression can be seen in all groups at smooth muscle cell cytoplasm of the media layer, and dense immunopositive reactions were seen in chlorpyrifos and control groups of aorta samples respectively. In conclusion, chlorpyrifos induced NO production in aorta following an increase in NOS expression.

## Figures and Tables

**Table 1:** The biochemical parameters in the plasma of the experimental groups.

Parameters	Control 1	Chlorpyrifos	Corn oil (control 2)	*P*
AChE (U/L)	815.37 ± 76.52^a^	474.52 ± 37.87^b^	788.64 ± 108.34^a^	0.009
CK (U/L)	100.45 ± 9.61	147.91 ± 18.52	133.20 ± 12.92	0.070
LDH (U/L)	750.25 ± 71.72^b^	1753.28 ± 252.08^a^	1097.25 ± 160.16^b^	0.002
AST (U/L)	107.65 ± 8.98^b^	142.53 ± 9.66^a^	115.43 ± 7.68^b^	0.024
ALT (U/L)	36.48 ± 2.82^a^	47.85 ± 2.04^b^	46.54 ± 8.23^b^	0.007
GGT (U/L)	9.42 ± 0.85^b^	14.44 ± 1.11^a^	11.19 ± 1.63^ab^	0.026
TP (mg/dL)	7.31 ± 0.21	7.50 ± 0.13	7.40 ± 0.17	0.752
Alb (mg/dL)	3.27 ± 0.12	3.60 ± 0.10	3.42 ± 0.09	0.107

^ab^Mean values within the same row with different superscripts are significantly different, *P* < 0.05, *n*: 10.

**Table 2:** The nitric oxide levels in the plasma and the aorta of the experimental groups.

Parameter	Control 1	Corn oil (control 2)	Chlorpyrifos
NO-aorta (*μ*mol/mg-protein)	2.18 ± 0.42^a^	1.99 ± 0.47^a^	3.11 ± 0.82^b^
NO-plasma (*μ*mol/mL)	0.28 ± 0.07^a^	0.22 ± 0.05^a^	0.43 ± 0.10^b^

^ab^Mean values within the same row with different superscripts are significantly different, *P* < 0.05, *n*: 8.

## References

[1] Milatovic D, Gupta RC, Aschner M (2006). Anticholinesterase toxicity and oxidative stress. *TheScientificWorldJournal*.

[2] Barr DB, Angerer J (2006). Potential uses of biomonitoring data: a case study using the organophosphorus pesticides chlorpyrifos and malathion. *Environmental Health Perspectives*.

[3] Pope CN (1999). Organophosphorus pesticides: do they all have the same mechanism of toxicity?. *Journal of Toxicology and Environmental Health B*.

[4] Goel A, Dani V, Dhawan DK (2005). Protective effects of zinc on lipid peroxidation, antioxidant enzymes and hepatic histoarchitecture in chlorpyrifos-induced toxicity. *Chemico-Biological Interactions*.

[5] Slotkin TA, Oliver CA, Seidler FJ (2005). Critical periods for the role of oxidative stress in the developmental neurotoxicity of chlorpyrifos and terbutaline, alone or in combination. *Developmental Brain Research*.

[6] Förstermann U (2010). Nitric oxide and oxidative stress in vascular disease. *Pflugers Archiv-European Journal of Physiology*.

[7] Violi F, Marino R, Milite MT, Loffredo L (1999). Nitric oxide and its role in lipid peroxidation. *Diabetes/Metabolism Research and Reviews*.

[8] Rajeswara Rao M, Kanji VK, Sekhar V (1999). Pesticide induced changes of nitric oxide synthase in rat brain in vitro. *Drug and Chemical Toxicology*.

[9] Chang AYW, Chan JYH, Kao FJ, Huang CM, Chan SHH (2001). Engagement of inducible nitric oxide synthase at the rostral ventrolateral medulla during mevinphos intoxication in the rat. *Journal of Biomedical Science*.

[10] Çetin N, Çetin E, Eraslan G, Bilgili A (2007). Chlorpyrifos induces cardiac dysfunction in rabbits. *Research in Veterinary Science*.

[11] Slotkin TA, Tate CA, Cousins MM, Seidler FJ (2005). Imbalances emerge in cardiac autonomic cell signaling after neonatal exposure to terbutaline or chlorpyrifos, alone or in combination. *Developmental Brain Research*.

[12] Gordon CJ, Padnos BK (2000). Prolonged elevation in blood pressure in the unrestrained rat exposed to chlorpyrifos. *Toxicology*.

[13] Smith EG, Gordon CJ (2005). The effects of chlorpyrifos on blood pressure and temperature regulation in spontaneously hypertensive rats. *Basic and Clinical Pharmacology and Toxicology*.

[14] Yavuz T, Delibas N, Yildirim B (2005). Vascular wall damage in rats induced by organophosphorus insecticide methidathion. *Toxicology Letters*.

[15] Katz LS, Marquis JK (1989). Modulation of central muscarinic receptor binding in vitro by ultralow levels of the organophosphate paraoxon. *Toxicology and Applied Pharmacology*.

[16] Bakry NMS, El-Rashidy AH, Eldefrawi AT, Eldefrawi ME (1988). Direct actions of organophosphate anticholinesterases on nicotinic and muscarinic acetylcholine receptors. *Journal of Biochemical Toxicology*.

[17] Lim SL, Sim MK, Loke WK (2000). Acetylcholinesterase-independent action of diisopropyl-flurophosphate in the rat aorta. *European Journal of Pharmacology*.

[18] Aly N, El-Gendy K, Mahmoud F, El-Sebae AK (2010). Protective effect of vitamin C against chlorpyrifos oxidative stress in male mice. *Pesticide Biochemistry and Physiology*.

[19] Mansour SA, Mossa ATH (2009). Lipid peroxidation and oxidative stress in rat erythrocytes induced by chlorpyrifos and the protective effect of zinc. *Pesticide Biochemistry and Physiology*.

[20] Verma RS, Mehta A, Srivastava N (2007). In vivo chlorpyrifos induced oxidative stress: attenuation by antioxidant vitamins. *Pesticide Biochemistry and Physiology*.

[21] Çetinkaya MA, Baydan E (2010). Investigation of in vitro effects of ethephon and chlorpyrifos, either alone or in combination, on rat intestinal muscle contraction. *Interdisciplinary Toxicology*.

[22] Aydın A, Orhan H, Sayal A, Özata M, Şahin G, Işımer A (2001). Oxidative stress and nitric oxide related parameters in type II diabetes mellitus: effects of glycemic control. *Clinical Biochemistry*.

[23] Macia RA, Matthews WD, Lafferty J, DeMarinis RM (1984). Assessment of alpha-adrenergic receptor subtypes in isolated rat aortic segments. *Naunyn-Schmiedeberg’s Archives of Pharmacology*.

[24] Ebeigbe AB, Campbell PI (1986). Inhibitory effect of dichlorvos on arterial smooth muscle contraction. *Pharmacological Research Communications*.

[25] Karagül H, Altıntaş A, Fidancı UR, Sel T (2000). Karaciğer fonksiyonları. *Klinik Biyokimya*.

[26] Mansour SA, Mossa ATH (2010). Oxidative damage, biochemical and histopathological alterations in rats exposed to chlorpyrifos and the antioxidant role of zinc. *Pesticide Biochemistry and Physiology*.

[27] Ncibi S, Ben Othman M, Akacha A, Krifi MN, Zourgui L (2008). *Opuntia ficus indica* extract protects against chlorpyrifos-induced damage on mice liver. *Food and Chemical Toxicology*.

[28] Mansour SA, Mossa AH (2005). Comparative effects of some insecticides as technical and formulated on male albino rats. *Journal of Egyption Society of Toxicology*.

[29] Bebe FN, Panemangalore M (2003). Exposure to low doses of endosulfan and chlorpyrifos modifies endogenous antioxidants in tissues of rats. *Journal of Environmental Science and Health B*.

[30] Mortensen SR, Hooper MJ, Padilla S (1998). Rat brain acetylcholinesterase activity: developmental profile and maturational sensitivity to carbamate and organophosphorus inhibitors. *Toxicology*.

[31] Wielgomas B, Krechniak J (2007). Effect of *α*-cypermethrin and chlorpyrifos in a 28-day study on free radical parameters and cholinesterase activity in wistar rats. *Polish Journal of Environmental Studies*.

[32] Guvenc Tuna B, Ozturk N, Comelekoglu U, Yilmaz BC (2011). Effects of organophosphate insecticides on mechanical properties of rat aorta. *Physiological Research*.

[33] Zhou JF, Xu GB, Fang WJ (2002). Relationship between acute organophosphorus pesticide poisoning and damages induced by free radicals. *Biomedical and Environmental Sciences*.

[34] Alp H, Aytekin I, Hatipoglu NK, Alp A, Ogun M (2012). Effects of sulforophane and curcumin on oxidative stress created by acute malathion toxicity in rats. *European Review For Medical and Pharmacological Sciences*.

[35] Soltaninejad K, Shadnia S, Afkhami-Taghipour M, Saljooghi R, Mohammadirad A, Abdollahi M (2007). Blood *β*-glucuronidase as a suitable biomarker at acute exposure of severe organophosphorus poisoning in human. *Human and Experimental Toxicology*.

[36] Kim YB, Hur GH, Shin S, Sok DE, Kang JK, Lee YS (1999). Organophosphate-induced brain injuries: delayed apoptosis mediated by nitric oxide. *Environmental Toxicology and Pharmacology*.

[37] Kanbur M, Liman BC, Eraslan G, Altinordulu S (2008). Effects of cypermethrin, propetamphos, and combination involving cypermethrin and propetamphos on lipid peroxidation in mice. *Environmental Toxicology*.

[38] Kaya S, Ünsal A, Kaya S, Pirinçci İ, Bilgili A (2000). İlaçların etkileri. *Veteriner Uygulamalı Farmakoloji*.

[39] Benzer F, Ozan S (2003). Fasciola hepatica ile enfekte koyunlarda lipid peroksidasyonu, antioksidan enzimler ve nitrik oksit düzeyleri. *Turkish Journal of Veterinary & Animal Sciences*.

[40] Kılınç A, Kılınç K (2003). *Nitrik oksit, biyolojik fonksiyonları ve toksik etkileri*.

[41] Tunçtan B, Abacıoğlu N (1998). Biyolojik örneklerde nitrik oksit ölçümü: diazotizasyon yöntemi. *Farmasötik Bilimler Dergisi*.

